# Tetanus and Diphtheria Seroprotection among Children Younger Than 15 Years in Nigeria, 2018: Who Are the Unprotected Children?

**DOI:** 10.3390/vaccines11030663

**Published:** 2023-03-15

**Authors:** Rania A. Tohme, Heather M. Scobie, Oyeladun Okunromade, Temitope Olaleye, Faisal Shuaib, Tunde Jegede, Ridwan Yahaya, Ndodo Nnaemeka, Bola Lawal, Abiodun Egwuenu, Nishanth Parameswaran, Gretchen Cooley, Qian An, Melissa Coughlin, Bassey B. Okposen, Ifedayo Adetifa, Omotayo Bolu, Chikwe Ihekweazu

**Affiliations:** 1 Global Immunization Division, Centers for Disease Control and Prevention, Atlanta, GA 30329, USA; 2 Nigeria Center for Disease Control, Abuja 900211, Nigeria; 3 Institute of Human Virology, FCT, Abuja 900211, Nigeria; 4 National Primary Healthcare Development Agency, Area 11, Garki, Abuja 900247, Nigeria; 5 Division of Parasitic Diseases and Malaria, Centers for Disease Control and Prevention, Atlanta, GA 30329, USA; 6 Division of Viral Diseases, Centers for Disease Control and Prevention, Atlanta, GA 30329, USA

**Keywords:** seroprevalence, tetanus, diphtheria, Nigeria, serology, vaccine preventable diseases

## Abstract

Serological surveys provide an objective biological measure of population immunity, and tetanus serological surveys can also assess vaccination coverage. We undertook a national assessment of immunity to tetanus and diphtheria among Nigerian children aged <15 years using stored specimens collected during the 2018 Nigeria HIV/AIDS Indicator and Impact Survey, a national cross-sectional household-based survey. We used a validated multiplex bead assay to test for tetanus and diphtheria toxoid-antibodies. In total, 31,456 specimens were tested. Overall, 70.9% and 84.3% of children aged <15 years had at least minimal seroprotection (≥0.01 IU/mL) against tetanus and diphtheria, respectively. Seroprotection was lowest in the north west and north east zones. Factors associated with increased tetanus seroprotection included living in the southern geopolitical zones, urban residence, and higher wealth quintiles (*p* < 0.001). Full seroprotection (≥0.1 IU/mL) was the same for tetanus (42.2%) and diphtheria (41.7%), while long-term seroprotection (≥1 IU/mL) was 15.1% for tetanus and 6.0% for diphtheria. Full- and long-term seroprotection were higher in boys compared to girls (*p* < 0.001). Achieving high infant vaccination coverage by targeting specific geographic areas and socio-economic groups and introducing tetanus and diphtheria booster doses in childhood and adolescence are needed to achieve lifelong protection against tetanus and diphtheria and prevent maternal and neonatal tetanus.

## 1. Introduction

Historically, vaccination coverage surveys have been used to assess the performance of immunization programs and identify areas at risk for vaccine-preventable diseases (VPDs). However, data have shown that VPD coverage surveys have limitations due to poor documentation of immunization history and parental recall bias [1]. In settings with weak surveillance or unreliable vaccination coverage, serological surveillance can potentially play an important role for appropriately directing interventions to improve population immunity [2,3]. Serological surveys are increasingly being used to guide immunization policy and strategy from support of vaccine introduction, evidence generation for optimizing timing of booster doses, to the verification of disease elimination [2,3,4,5,6,7,8,9,10,11,12,13,14]. In the case of tetanus, serological surveys can also assess routine vaccination coverage as tetanus infection does not lead to development of protective antibodies [14]. Tetanus serosurveys can identify areas or subgroups yet to be reached with routine immunization and also assess duration of vaccine-induced immunity to inform introduction of tetanus (and diphtheria) booster doses [14]. Depending on the disease, serum antibody levels can be maintained for years following a person’s vaccination or exposure to a pathogen, like the one that causes diphtheria; therefore, specimens collected during cross-sectional surveys contain an immense amount of information about current and past pathogen exposure and levels of immunity [4].

High tetanus burden in Nigeria has been documented [15], with the tetanus case fatality ratio (CFR) estimated at 43% [16]. In addition, high diphtheria burden and recurring diphtheria outbreaks with high CFR have been documented in Nigeria, including a recent outbreak in 2023 [17,18,19]. In 2011, over 60% of diphtheria cases occurred among children younger than 10 years of age and over 95% were unvaccinated [17]. Nigeria’s immunization schedule includes three doses of diphtheria-tetanus-pertussis (DTP) containing vaccine at 6, 10, and 14 weeks of age. However, diphtheria and tetanus immunity wane over time and by school age (5–6 years old), many children are susceptible to infection. For this reason, in addition to the three primary DTP (DTP3) doses given before the age of one year, WHO recommends three booster doses of tetanus- and diphtheria-containing vaccines be provided to children and adolescents at the ages of 12–23 months, 4–7 years and 9–15 years to provide protection across the life-course [20,21].

Data on tetanus and diphtheria immunity among children in Nigeria are needed to evaluate susceptibility to VPDs and for use in program improvement to prevent tetanus cases and diphtheria epidemics. We conducted a national serological assessment to estimate immunity to tetanus and diphtheria among Nigerian children born during 2004–2018 (aged <15 years old at the time of the survey in 2018) and by subpopulation (age, sex, zone, state, urban/rural, and wealth quintile) to identify immunity gaps and specific populations that might need targeted interventions to improve vaccination coverage.

## 2. Materials and Methods

### 2.1. Study Population and Sampling

The target population included children younger than 15 years of age and residing in Nigeria. We used stored specimens collected during the 2018 Nigeria HIV/AIDS Indicator and Impact Survey (NAIIS) (https://www.naiis.ng/ (accessed on 15 January 2023). NAIIS was a national cross-sectional, household-based survey with a multi-stage cluster sampling design. In the first stage, clusters (enumeration areas) were selected using projected census data. Within each selected enumeration area, households were chosen through systematic sampling. Children less than 15 years of age were sampled in every 4th household (28,220 households sampled for inclusion of children aged 0–14 years). The eligibility criteria for children included having resided in the selected household or spent the night in that household before the survey; parents or guardians willing to provide written informed consent/permission in English, Hausa, Yoruba, or Igbo; and for children aged 10–14 years, the child is able and willing to provide written assent in English, Hausa, Yoruba, or Igbo. Further information on NAIIS methodology is available [22].

Assuming the most conservative estimate of 50% seroprevalence for tetanus and diphtheria with a 95% confidence interval, a design effect of 2, and a 5% non-response rate (e.g., not enough specimen), a sample size of 810 children per state (270 children each in age groups 0–4 years, 5–9 years and 10–14 years) would produce a precision of +/−9% for state- and age-level estimates. In total, 30,000 children would be needed from all 36 states and Abuja Federal Capital Territory (FCT) (10,000 specimens per age group). NAIIS collected specimens from 32,480 children ages 0–14 years, of which 32,337 (99%) assented for storage and future testing of specimens and were included in this study.

### 2.2. Laboratory Methods and Testing

From each participant, we used 6 mm sections punched from dried blood spots collected on Whatman filter paper corresponding to 5 µL of serum to test for tetanus and diphtheria IgG on a multiplex bead assay platform. An additional 6 mm section was used in 10% of the samples to repeat testing for quality control.

#### 2.2.1. Antigens and Couplings

Tetanus toxoid was purchased from Massachusetts Biological Laboratories (Boston, MA, USA) and diphtheria toxoid was purchased from List Biological Laboratories (Campbell, CA, USA). Antigens were coupled to MagPlex microspheres (Luminex Corp, Austin, TX, USA), as described previously [5,23], using antigen concentrations of 12.5 µg/12.5 × 10^6^ microspheres for tetanus toxoid and 60 µg/12.5 × 10^6^ microspheres for diphtheria toxoid.

#### 2.2.2. Sample Preparation and Bead Assay

Samples were diluted to a final serum concentration of 1:400 in Buffer B (PBS pH 7.2 plus 0.5% casein, 0.8% PVP, 0.5% PVA, 0.3% Tween 20, 0.02% sodium azide and 3 µg/mL of Escherichia coli lysate containing GST). All incubation steps were performed at room temperature in 50 µL reaction volumes protected from light while shaking at 600 rpm. Incubation steps were followed by three washes with 200 µL PBS pH 7.2 containing 0.05% Tween 20 (PBST) using a handheld magnet. Diluted samples were incubated with 625 microspheres/antigen/well for 90 min, followed by 45 min incubation with secondary antibodies at a concentration of 50 ng/well for anti-human IgG and 40 ng/well for anti-IgG4 (Southern Biotech, Birmingham, AL, USA). Incubations with 250 ng/well streptavidin-R phycoerythrin (Invitrogen, Waltham, MA, USA) and assay buffer alone were carried out, as previously described [5]. Beads were resuspended in 100 µL PBS pH 7.2 and stored overnight at 4 °C prior to reading on MAGPIX (Luminex Corporation, Austin, TX, USA).

#### 2.2.3. Quality Control and Assurance

Data were output as median fluorescence intensity (MFI). To control for background reactivity, each assay included 2 blank wells containing Buffer B only. Samples were run in singlicate. Each plate also contained 1 negative serum control and 2 positive serum controls run in duplicate, as well as a positive pool serum control that was serially diluted to make an 8-point curve to cover the linear range of MFI for most antigens. Samples and controls were analyzed with the average of the plate background subtracted (MFI-BG). As a measure of assay-to-assay variation, the average reactivities of the antigens with the controls was used to create criteria for accepting or rejecting plate data.

#### 2.2.4. Cutoffs and Interpolation

WHO international standards TE-3 and 10/262 were used to convert MFI-BG for tetanus and diphtheria, respectively, to IU/mL. Dilutions of each standard were run on separate plates over the course of the study at approximately one-month intervals, with a total of seven individual runs averaged to create a single curve per antigen for all samples. Curve fitting and interpolation were performed in GraphPad Prism v.9 using 5PL non-linear regression. Conversion of values to IU/mL was conducted in SAS.

Tetanus and diphtheria antibody seroprotection were defined using the standard cut-off of 0.01 IU/mL, which corresponds to the minimum level of antibody required for protection against tetanus and diphtheria [20,21,24]. Tetanus IgG testing on MBA has been previously validated against the reference standard double antigen ELISA IgG with a sensitivity of 99% and specificity of 92% at the ≥0.01 IU/mL cutoff [5]. Diphtheria IgG testing on MBA was validated against the Vero cell neutralization assay IgG with a sensitivity of 95% and specificity of 83% at the ≥0.01 IU/mL cutoff for minimal protection [23]. Increasing antibody concentrations against tetanus are associated with decreased risk of infection and increased duration of protection [25], and tetanus antibody concentrations ≥1 IU/mL are typically associated with long-term protection [6,25]. Tetanus and diphtheria antibody seroprotection were further categorized as IgG < 0.01 IU/mL (lack of protection), 0.01–<0.1 IU/mL (minimal protection), 0.10–<1 IU/mL (full protection), and ≥1 IU/mL (long-term protection) [5,6,23,24,25,26].

### 2.3. Statistical Analyses

Statistical analyses followed the WHO tetanus serosurvey guidance published in 2018 [14]. Estimates of tetanus and diphtheria seroprotection (at ≥0.01, ≥0.1 and ≥1.0 IU/mL antibody levels) and Wilson 95% confidence intervals were calculated using sample weights and a Taylor series linearization method to account for survey design. To assess risk factors, seroprotection was calculated by sociodemographic characteristics (age, sex, zone, state, urban/rural, and wealth quintile) and associations were assessed using Rao-Scott chi-square tests. A *p*-value of <0.05 was considered statistically significant. To assess waning immunity and the need for tetanus–diphtheria booster dose introduction, estimates of the proportions of children by age and different antibody level categories for tetanus and diphtheria (<0.01, 0.01 to 0.099, 0.1 to 0.99, and ≥1.0 IU/mL) were calculated; geometric mean antibody levels and 95% confidence intervals (95%CI) were also calculated accounting for the survey design. We assessed the contribution of routine immunization, waning immunity, and natural exposure (for diphtheria) by assessing percentage of children seroprotected (≥0.01 IU/mL) against diphtheria (including through natural infection) but not against tetanus (<0.01 IU/mL) by age group (4–11 months, 12–23 months, 24–35 months, 36–47 months, and 48–59 months; 0–4, 5–9 and 10–14 years). All statistical analyses were conducted using SAS v9.4 (SAS Institute, Inc., Cary, NC, USA).

Finally, we triangulated data on the proportion of children with minimal and full seroprotection to tetanus by age with vaccination coverage to assess comparability between serosurvey results and vaccination coverage results in those age groups given that tetanus immunity can only be achieved by vaccination. For this purpose, we used DTP3 coverage among children 12–23 months of age reported from three data sources that covered the birth cohorts included in this survey (born during 2004–2018): (1) WHO/UNICEF estimates of national immunization coverage (WUENIC); (2) country-reported official estimates for DTP3 coverage for each birth cohort [27]; and (3) when available for the birth cohorts included in this survey, we also used vaccination coverage survey estimates of DTP3 coverage among children 12–23 months from Demographic and Health Surveys (DHS) (for 2018, 2013, and 2008) [28] and Multiple Indicator Cluster Surveys (MICS) (for 2016, 2011, and 2007) [29].

## 3. Results

### 3.1. General Demographics of Study Population

Of the 32,337 available specimens from children aged 0–14 years, 31,456 (97%) specimens yielded results for tetanus and diphtheria immunity. A slightly higher percentage of 5–9-year-olds (39.5%) compared to younger (30.2%) and older (30.3%) age groups, and an almost equal proportion of males (51.0%) and females (49.0%) were tested. The highest proportion of participants tested was in the north west geopolitical zone (27.9%) compared to almost equal proportions in other zones (range: 12.7–16.0%). Rural areas accounted for a higher proportion of specimens tested (58.6%) than urban areas (41.4%) (Table 1).

### 3.2. Tetanus and Diphtheria Seroprotection

Overall, 70.9% (95% confidence interval (CI): 69.9–72.0%) of children aged <15 years had at least minimal seroprotection (≥0.01 IU/mL) against tetanus and 84.3% (95%CI: 83.6–85.0%) had at least minimal seroprotection (≥0.01 IU/mL) against diphtheria (Table 2). Full seroprotection (≥0.1 IU/mL) was 42.2% (95% CI: 41.2–43.3%) for tetanus and 41.7% (95% CI: 40.9–42.5%) for diphtheria; long-term seroprotection (≥1.0 IU/mL) was 15.1% (95% CI: 14.5–15.7%) for tetanus and 6.0% (95% CI: 5.7–6.4%) for diphtheria (Table 2 and Table S1).

The proportion of children aged <15 years with seroprotection against tetanus and diphtheria increased with age (*p* < 0.001). While minimal tetanus seroprotection was not different between male and female children, full- and long-term tetanus and diphtheria seroprotection were higher in male compared to female children (Table 2 and Table S1). Tetanus and diphtheria minimal, full, and long-term seroprotection levels were lowest in the north west and north east geopolitical zones, while the south east and south south geopolitical zones had the highest tetanus and diphtheria seroprotection levels (Table 2 and Table S1).

**Figure 1:**
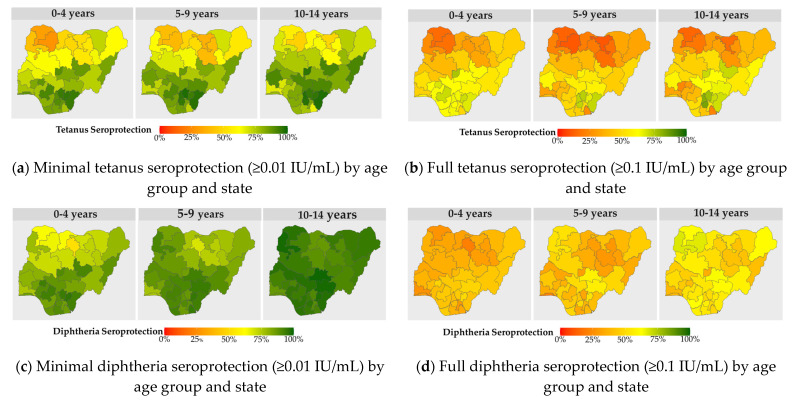
Tetanus and diphtheria minimal and full seroprotection among children aged <15 years by age group and state, Nigeria, 2018 (**a**–**d**).

Tetanus and diphtheria seroprotection varied significantly by state and age group (Figure 1). The proportion of children with minimal tetanus seroprotection among children aged 0–4 years was at least 90% in Enugu, Ebonyi, Cross-river, and Edo states in the south east and south south zones. While most of the states in the north east and north west geopolitical zones had very low seroprotection against tetanus, three states, Adamawa, Taraba, and Yobe, had higher minimal tetanus seroprotection (>70%) among children aged 0–4 years in these zones. Only three states, FCT Abuja, Edo, and Ebonyi, had >70% of children aged 0–4 years with full tetanus seroprotection; however, seven states had >70% full tetanus seroprotection among children aged 10–14 years (Figure 1b). It is noticeable that tetanus seroprotection was higher among children aged 10–14 years compared with children aged 5–9 years in some states in the north west and north east geopolitical zones despite the lack of childhood Td booster doses in the national immunization program. The proportion of children with diphtheria seroprotection increased with age across the majority of the states (Figure 1c,d). While Sokoto, Zamfara, and Jigawa in the north west zone had less than 50% of children aged 10–14 years who had minimal seroprotection against tetanus, those states had greater than 90% of children with minimal diphtheria seroprotection (Figure 1a,c).

**Figure 2:**
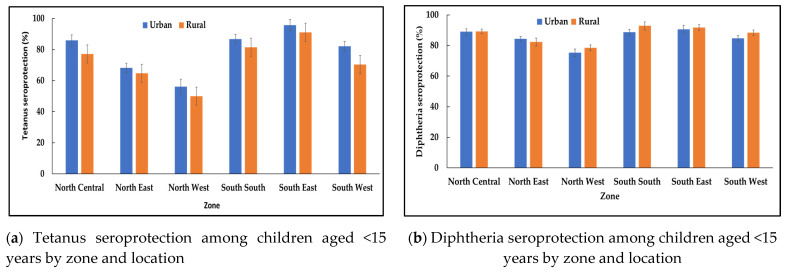
Tetanus and diphtheria minimal seroprotection (≥0.01 IU/mL) among children aged <15 years by zone and location, Nigeria, 2018 (**a**,**b**).

The proportion of children <15 years with minimal or full tetanus seroprotection was higher in urban compared to rural areas (Table 2) and was statistically significantly different in all geopolitical zones (*p* < 0.05) except in the north east zone (Figure 2a). The largest discrepancy between urban and rural areas with regard to proportion of children seroprotected against tetanus was in the south west zone (Figure 2a). In comparison, the proportion of children <15 years with minimal or full diphtheria seroprotection was higher in rural compared to urban areas (*p* < 0.05) (Table 2). However, no significant difference was observed in the proportion of children who were seroprotected against diphtheria between rural and urban areas by geopolitical zone except for the south south and south west zones, where the proportion seroprotected was higher in rural areas (*p* < 0.05) (Figure 2b).

**Figure 3:**
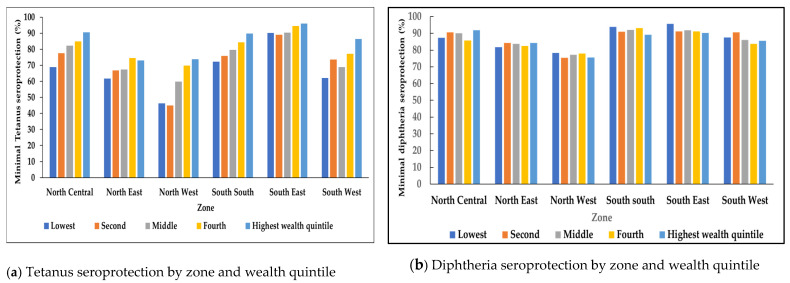
Tetanus and diphtheria at least minimal seroprotection (≥0.01 IU/mL) among children aged <15 years by zone and wealth quintile, Nigeria, 2018 (**a**,**b**).

The proportion of children <15 years who had minimal seroprotection against tetanus was higher in more wealthy quintiles, a pattern that was significant across all geopolitical zones (Figure 3a). In comparison, the proportion of children <15 years who had minimal seroprotection against diphtheria did not show a specific trend by wealth quintile. A significant difference in the proportion of children <15 years with minimal diphtheria seroprotection was observed by wealth quintile in the north central and south south geopolitical zones (*p* < 0.01) (Figure 3b).

Among children aged <5 years, the proportion of children with minimal seroprotection for tetanus and diphtheria was 77.7% for children aged 4–11 months, 72.5% for 12–23 months, 66.2% for 24–35 months, 59.4% for 36–47 months, and 55.2% for 48–59 months, while the proportions of those children without seroprotection to both tetanus and diphtheria were 12.4%, 17.9%, 20.8%, 19.6%, and 19.0%, respectively. The proportion of children who had minimal seroprotection against diphtheria but not against tetanus increased with age from 5.8% among children aged 4–11 months, to 6.6% for 12–23 months, 9.4% for 24–35 months, 16.3% for 36–47 months, and 20.2% for 48–59 months. Overall, 13.2% of children aged 0–4 years, 20.7% of children aged 5–9 years, and 21.6% of children 10–14 years had minimal seroprotection to diphtheria but no seroprotection to tetanus.

**Figure 4:**
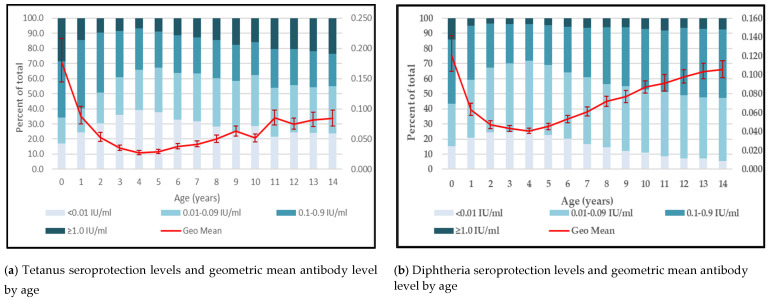
Tetanus and diphtheria seroprotection levels and geometric mean antibody level by age, Nigeria, 2018 (**a**,**b**).

By one-year age cohort, the proportion of children who were seroprotected against tetanus was lowest in children aged 2–10 years old, and waning immunity to tetanus was observed starting at the age of 3 years based on the drop in geometric mean antibody level (Figure 4a). The proportion of children with long-term tetanus seroprotection was highest in children aged younger than 1 year followed by 11–14 year-olds which corresponded to a similar trend in geometric mean antibody levels (Figure 4a). In comparison, geometric mean antibody levels and the proportions of children seroprotected against diphtheria were lowest among children aged 2–5 years but increased gradually among children aged 6–14 years (Figure 4b).

### 3.3. Triangulation of Tetanus Seroprotection and Vaccination Coverage

**Figure 5:**
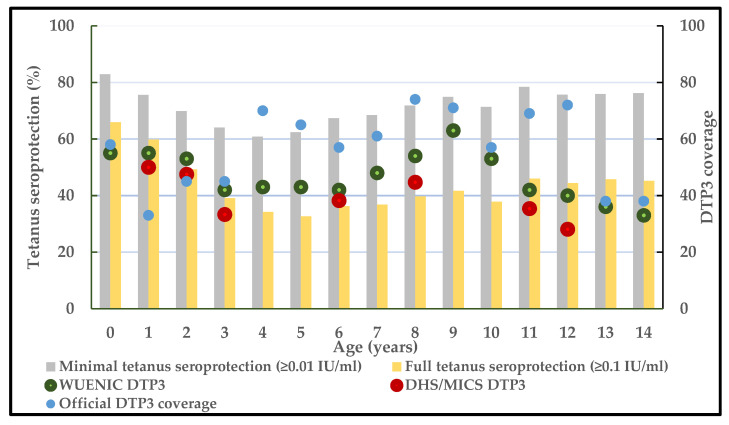
Triangulation of tetanus seroprotection and DTP3 vaccination coverage among children aged <15 years, Nigeria, 2018. WUENIC: WHO/UNICEF estimates of national immunization coverage; DTP3: 3 doses of diphtheria-tetanus-pertussis vaccine; DHS: Demographic and Health Survey; MICS: Multiple Indicator Cluster Survey.

The proportions of children with minimal tetanus seroprotection (≥0.01 IU/mL) were higher than annual estimates of DTP3 coverage from WHO/UNICEF estimates of national vaccination coverage (WUENIC) and vaccination coverage surveys (DHS/MICS) in all age cohorts but lower than official country-reported DTP3 coverage among children aged 4, 5 and 8 years (Figure 5). When available, estimates of DTP3 coverage from DHS or MICS surveys correlated better with the proportion of children who had full seroprotection against tetanus compared with minimal seroprotection.

## 4. Discussion

This serological assessment using specimens previously collected in 2018 from over 30,000 children aged less than 15 years provided the first estimates of tetanus and diphtheria seroprotection among children in Nigeria. Overall, both tetanus and diphtheria seroprotection were below the recommended vaccination coverage levels of at least 80% for tetanus [25] and 90% for diphtheria [26] to prevent infections in the population, especially among younger age groups. Full and long-term seroprotection against tetanus and diphtheria were low across all age groups and geographies. The survey also identified demographic and socio-economic factors that were associated with decreased seroprotection to tetanus and diphtheria. Tetanus seroprotection varied significantly by age, sex, geopolitical zone, state, urban/rural, and wealth quintile. Diphtheria seroprotection varied mainly by age, sex, geopolitical zone, and state.

Comparisons of tetanus seroprotection by age showed that children aged 2–5 years had the highest proportions of children who lacked tetanus seroprotection and the lowest geometric mean antibody levels; this finding correlates with studies reporting decreases in tetanus seroprotection starting at the age of 2 years [25]. Similarly, diphtheria seroprotection and geometric mean antibody levels were lowest in children who were 2–6 years old. When comparing tetanus and diphtheria seroprotection by age group and state, we observed that proportions of children with diphtheria seroprotection among older age groups were highest in the areas that had the lowest tetanus seroprotection (Figure 1), which highlighted the important contribution of natural infection to diphtheria seroprotection in children ages five years and older. For example, Kano State had one of the lowest minimal seroprotection levels against tetanus (49%) and diphtheria (52%) among children aged <4 years, reflecting low routine immunization coverage, yet diphtheria immunity reached 87% in children aged 10–14 years. Similar observations were noted in Borno and other northern states, likely reflecting the frequent occurrence of diphtheria outbreaks mainly in Northern Nigeria [17,18]. The most recent outbreak in Kano during 2022–2023 caused over 25 deaths, mostly among children [19]. Hence, in addition to improving DTP3 vaccination coverage, Nigeria needs to consider introducing the WHO-recommended booster doses for tetanus and diphtheria at the ages of 12–23 months, 4–7 years, and 9–15 years to ensure long-term protection across the life-course and prevent recurring diphtheria outbreaks, as well as sustain maternal and neonatal tetanus elimination [20,21].

Given that tetanus seroprotection is an indirect measure of DTP3 vaccination coverage especially in younger age groups, tetanus seroprotection levels highlighted areas and populations requiring urgent attention to improve vaccination coverage. Girls had significantly lower levels of full and long-term seroprotection to tetanus and diphtheria compared to boys, indicating the need to ensure that girls are reached with immunization services and are adequately protected against VPDs. Gender-based inequity in access to vaccination services and protection from VPDs has been noted in multiple countries with associations in some cases with maternal education [11,30]. In Nigeria, higher maternal education was associated with higher childhood vaccination coverage based on analysis of the 2018 DHS [31] and with higher utilization of essential maternal and child health services based on analysis of five national household surveys [32].

Geographic variations in tetanus and diphtheria seroprotection highlighted zones and states that require remediation to decrease the number of children susceptible to VPDs. The north west and north east geopolitical zones had the lowest proportions of tetanus and diphtheria seroprotection indicating the need for catch-up vaccination as well as strengthening infant immunization coverage. However, heterogeneity in seroprotection among children aged 0–4 years was also observed between states within the same geopolitical zone reflecting variation in routine immunization coverage. For example, Adamawa state in the north east had >80% minimal seroprotection against tetanus and diphtheria among children aged 0–4 years, indicating better routine immunization performance relative to the north east zone, which had <70% tetanus seroprotection overall. Similarly, inequities in seroprotection against tetanus were also observed in states located in the south south and south east geopolitical zones which were better performing overall than other geopolitical zones. Therefore, serosurvey results highlighted subnational geographies that would benefit from intensive support to improve vaccination coverage and remediations efforts to prevent diphtheria outbreaks. Our findings were similar to a mapping of areas at risk for measles in Nigeria, which indicated that susceptibility to multiple VPDs tended to cluster in similar states and geopolitical zones [33]. Inequities in seroprotection against tetanus were noted in rural versus urban areas and lower versus higher wealth quintiles. These factors have been shown to be associated with tetanus seroprotection, vaccination coverage or access to maternal and child health services in Nigeria and other countries [11,30,31,32,34,35].

Triangulating different data sources enabled further interpretation of serosurvey findings. When comparing seroprevalence and vaccination coverage data, DTP3 coverage underestimated seroprotection to tetanus across children of all ages. Immunity can result from a partial series of a multidose vaccine (i.e., DTP2) [14]. Vaccination coverage surveys are also at risk of information and recall bias, and both card documentation and recall have been found to underestimate actual vaccination coverage [9]. The limitations of measuring vaccination coverage based on card or recall have been noted in other tetanus serosurveys [5,7,10,11]. We observed increased tetanus seroprotection among children aged 10–14 years compared with those aged 5–9 and 0–4 years, unlike the gradual decrease in tetanus seroprotection with age documented in other countries related to waning immunity and not providing childhood booster doses against tetanus and diphtheria [8,11,12]. The increased tetanus seroprotection in children aged 10–14 years and the gradual increase in geometric mean antibodies starting at the age of 6 years in Nigeria is most likely a result of the multiple meningitis vaccination campaigns targeting ages 1–29 years in states at high-risk for meningitis in Northern Nigeria during 2011–2014 [36,37]. These campaigns used the meningococcal A conjugate vaccine (MACV) which is conjugated to tetanus toxoid [38] and has been shown to boost tetanus immunity in other countries in the African meningitis belt [39,40]. A tetanus serosurvey conducted in Mali before and after the MACV campaigns showed increase in geometric mean concentrations and tetanus immunity among people aged 1–29 years from 57% to 88% [39]. In addition, clinical studies on MACV showed robust tetanus serologic response in people aged 1–29 years after MACV vaccination [40]. Therefore, the four rounds of MACV campaigns in 17 states in northern Nigeria might have helped boost tetanus immunity and contribute to the higher proportion of minimal, full, and long-term tetanus seroprotection in older children compared to younger age groups.

This serologic assessment used existing stored specimens from NAIIS, which was designed and implemented to make the survey as representative as possible. However, any deviations from protocol implementation may limit generalizability of the findings to the Nigerian population. For example, 72 (1.8%) enumeration areas out of a total of 4035 selected for NAIIS were unable to be visited because of security challenges and one area was not visited due to flooding, potentially limiting representativeness of survey estimates in these areas. The response rate for blood collection in children aged 0–9 years (68.5%) was lower than in children aged 10–14 years (92.3%), and while the distribution of children whose parents refused use of specimens for other tests was similar across gender, age, zone and cluster compared to those who were tested for tetanus and diphtheria, more of these children were in Lagos (15.6%) and Kano (11.0%) [22]. However, the survey included a large sample size which allowed for precise estimates of seroprotection down to the state level, and the overall population distribution of the NAIIS survey was similar to the population of Nigeria [41]. Finally, while the serosurvey results represent a specific time point (2018), results are still relevant in 2023 as vaccination coverage in Nigeria has not changed significantly compared to 2018. DTP3 coverage estimates were 56% during 2019–2021 compared to 55% in 2018 based on WUENIC estimates [27] and 57% in the MICS 2021 compared to 50% in the 2018 DHS [28,42]. These vaccination coverage estimates were still below the seroprotective levels needed to prevent infections. Hence, results in this serosurvey are still relevant to inform public health interventions and policy. In the future, it would be beneficial if testing for vaccine-preventable diseases could be integrated in parallel with other disease-specific testing in large population-based surveys, such as NAIIS, instead of waiting until completion of testing for other diseases to be able to access the specimens. This would enable timely availability of data showing granular differences in seroprotection in specific demographic and socio-economic groups and would help inform immediate targeted public health actions to address inequities in immunization.

## 5. Conclusions

This study provided the first estimates of tetanus and diphtheria seroprotection in Nigeria. The findings highlighted the need to target specific geographic areas and to consider gender and socio-economic equity in improving access and demand for vaccinations, as well as the need to introduce tetanus and diphtheria containing booster doses at 12–23 months, 4–7 years, and 9–15 years of age to address waning immunity and prevent tetanus cases and diphtheria outbreaks. The survey results also highlighted the benefit of supplementing vaccination coverage data with seroprevalence data and other data sources to provide a better assessment of the vaccination program and to help identify immunity gaps.

## Figures and Tables

**Table 1:** Demographic characteristics of children tested for tetanus and diphtheria, Nigeria, 2018.

	Number (N = 31,456)	Percentage
**Age group**		
0–4	9487	30.2
5–9	12,435	39.5
10–14	9534	30.3
**Sex**		
Female	15,425	49.0
Male	16,031	51.0
**Geopolitical zone**		
North central	4634	14.7
North east	5045	16.0
North west	8784	27.9
South south	4387	13.9
South east	4000	12.7
South west	4606	14.6
**Location**		
Urban	13,008	41.4
Rural	18,448	58.6

**Table 2:** Tetanus and diphtheria seroprotection among children aged <15 years, Nigeria, 2018.

		Tetanus ≥ 0.01 IU/mL			Tetanus ≥ 0.1 IU/mL			Diphtheria ≥ 0.01 IU/mL			Diphtheria ≥ 0.1 IU/mL		
	N	n	Percentage (95% CI)	*p*-Value	n	Percentage (95% CI)	*p*-Value	n	Percentage (95% CI)	*p* Value	n	Percentage (95% CI)	*p* Value
**Overall**	31,456	22,727	70.9 (69.9–72.0)	-	13,698	42.2 (41.2–43.3)	-	26,702	84.3 (83.6–85.0)	-	13,200	41.7 (40.9–42.5)	-
**Age group (years)**													
0–4	9487	6663	68.4 (66.9–70.0)	<0.0001	4579	46.4 (44.8–48.0)	<0.0001	7484	77.2 (75.8–78.6)	<0.0001	3472	35.0 (33.7–36.3)	<0.0001
5–9	12,435	8793	68.5 (67.1–69.8)		4863	37.0 (35.8–38.3)		10,425	82.5 (81.5–83.4)		4920	38.4 (37.3–39.6)	
10–14	9534	7271	75.4 (74.2–76.6)		4256	43.8 (42.4–45.2)		8793	92.1 (91.3–92.8)		4808	50.5 (49.2–51.7)	
**Sex**													
Female	15,425	11,147	70.9 (69.7–72.1)	0.9231	6473	40.6 (39.4–41.8)	<0.0001	12,920	83.0 (82.1–83.9)	<0.0001	5943	38.0 (37.1–39.0)	<0.0001
Male	16,031	11,580	71.0 (69.8–72.1)		7225	43.8 (42.6–45.0)		13,782	85.6 (84.7–86.3)		7257	45.2 (44.1–46.2)	
**Zone**													
North central	4634	3718	79.8 (77.3–82.1)	<0.0001	2463	53.5 (50.6–56.3)	<0.0001	4116	89.2 (87.9–90.4)	<0.0001	2134	46.7 (44.6–48.8)	<0.0001
North east	5045	3359	65.8 (63.2–68.2)		1920	37.3 (34.9–39.9)		4139	83.0 (81.5–84.5)		1984	40.6 (38.6–42.6)	
North west	8784	4608	52.8 (50.5–55.1)		2326	26.6 (24.6–28.7)		6870	77.1 (75.5–78.6)		3593	40.3 (38.6–42.0)	
South south	4387	3648	83.1 (81.2–84.8)		2176	49.2 (47.0–51.5)		3999	91.6 (90.7–92.5)		1951	45.2 (43.3- 47.2)	
South east	4000	3716	93.0 (91.8–94.0)		2707	68.0 (65.8–70.1)		3640	91.3 (90.2–92.2)		1810	45.6 (43.8–47.5)	
South west	4606	3678	79.6 (77.9–81.3)		2106	45.7 (43.7–47.7)		3938	85.5 (84.2–86.7)		1728	36.6 (34.8–38.4)	
**Location**													
Urban	13,008	9894	74.4 (72.7–76.1)	<0.0001	6247	46.1 (44.5–47.9)	<0.0001	10,893	83.0 (81.8–84.1)	0.0026	5034	38.4 (37.2–39.6)	<0.0001
Rural	18,448	12,833	68.2 (66.7–69.6)		7451	39.1 (37.7–40.6)		15,809	85.4 (84.4–86.3)		8166	44.3 (43.2–45.5)	

CI: confidence interval.

## Data Availability

Data are unavailable due to privacy consideration as datasets include global positioning system coordinates which might enable identification of location of study subjects. Only restricted individuals had access to the datasets for analyses purposes only.

## Supplementary Materials

The following supporting information can be downloaded at: https://www.mdpi.com/article/10.3390/vaccines11030663/s1, Table S1: Long term tetanus and diphtheria seroprotection among children aged <15 years, Nigeria 2018.
