# Supplementary material for: Tetanus and Diphtheria Seroprotection among Children Younger Than 15 Years in Nigeria, 2018: Who Are the Unprotected Children?
Source: Vaccines (Basel). 2023 Mar 15;11(3):663. doi: 10.3390/vaccines11030663 (PMC10056928; doi:10.3390/vaccines11030663)
Supplement: Supplementary file 1 [file vaccines-11-00663-s001.zip › vaccines-2255236-supplementary.pdf]

Table S1. Long term tetanus and diphtheria seroprotection among children aged <15 years, Nigeria 2018

		**Tetanus ≥ 1 IU/ml**		**Diphtheria ≥ 1 IU/ml**	
	**N**	**Percentage (95%CI)**	**p-value**	**Percentage (95%CI)**	**p-value**
**Overall**	31456	15.1 (14.5-15.7)		6.0 (5.7-6.4)	
**Age group**					
0 - 4	9487	11.6 (10.9-12.4)	<0.0001	5.2 (4.7-5.7)	<0.0001
5 - 9	12435	12.7 (11.9-13.5)		5.7 (5.2-6.2)	
10 - 14	9534	20.4 (19.3-21.4)		7.1 (6.5-7.7)	
**Sex**					
Female	15425	13.5 (12.8-14.2)	<0.0001	5.5 (5.1-5.9)	<0.001
Male	16031	16.6 (15.8-17.4)		6.5 (6.1-7.0)	
**Zone**					
Northcentral	4634	20.6 (18.9-22.4)	<0.0001	7.0 (6.1-7.9)	<0.001
Northeast	5045	12.4 (11.1-13.9)		4.7 (4.0-5.6)	
Northwest	8784	8.9 (7.8-10.0)		5.9 (5.3-6.6)	
South-south	4387	16.1 (14.8-17.6)		7.1 (6.3-8.1)	
Southeast	4000	29.5 (27.6-31.5)		6.1 (5.3-7.0)	
Southwest	4606	15.0 (13.6-16.5)		5.9 (5.1-6.8)	
**Location**					
Urban	13008	17.4 (16.4-18.5)	<0.0001	6.0 (5.5-6.5)	0.91
Rural	18448	13.2 (12.5-14.0)		6.0 (5.6-6.5)	
